# A clinical evaluation of two central venous catheter stabilization systems

**DOI:** 10.1186/s13613-019-0519-6

**Published:** 2019-04-17

**Authors:** Tarja J. Karpanen, Anna L. Casey, Tony Whitehouse, Jean-Francois Timsit, Olivier Mimoz, Mercedes Palomar, Tom S. J. Elliott

**Affiliations:** 1 Department of Clinical Microbiology, University Hospitals Birmingham NHS Foundation Trust, Queen Elizabeth Hospital, Mindelsohn Way, Edgbaston, Birmingham B15 2WB UK; 2 Department of Anaesthetics and Intensive Care, University Hospitals Birmingham NHS Foundation Trust, Queen Elizabeth Hospital, Edgbaston, B15 2WB UK; 3 APHP, Medical and infectious diseases ICU, Bichat Hospital, 75018 Paris, France; 4 Descision Science in Infectious Diseases, IAME U 1137 Inserm/Université Sorbonne-Paris-Cité, 75018 Paris, France; 5 Service des Urgences – SAMU 86, CHU de Poitiers, 6021 Poitiers Cedex, France; 6 Servicio de Medicina Intensiva, Hospital Universitari Arnau de Vilanova de Lleida, Av. Rovira Roure, 80, 25198 Lleida, Spain; 7 Corporate Division, University Hospitals Birmingham NHS Foundation Trust, Queen Elizabeth Hospital, Mindelsohn Way, Edgbaston, Birmingham B15 2WB UK; 8 INSERM U1070, Université de Poitiers, 86021 Poitiers Cedex, France

**Keywords:** Catheter migration, CVC, Securement, Suture, Sutureless

## Abstract

**Background:**

Central venous catheters (CVCs) are commonly secured with sutures which are associated with microbial colonization and infection. We report a comparison of a suture-free system with standard sutures for securing short-term CVC in an international multicentre, prospective, randomized, non-blinded, observational feasibility study. Consented critical care patients who had a CVC inserted as part of their clinical management were randomized to receive either sutures or the suture-free system to secure their CVC. The main outcome measures were CVC migration (daily measurement of catheter movement) and unplanned catheter removals.

**Results:**

The per cent of unplanned CVC removal in the two study groups was 2% (suture group 2 out of 86 patients) and 6% (suture-free group 5 out of 85 patients). Both securement methods were well tolerated in terms of skin irritation. The time and ease of application and removal of either securement systems were not rated significantly different. There was also no significant difference in CVC migration between the two securement systems in exploratory univariate and multivariate analyses. Overall, 42% (36 out of 86) of the CVC secured with sutures and 56% (48 out of 85) of the CVC secured with the suture-free securement system had CVC migration of ≥ 2 mm.

**Conclusions:**

The two securement systems performed similarly in terms of CVC migration and unplanned removal of CVC; however, the feasibility study was not powered to detect statistically significant differences in these two parameters.

**Trial registration:**

ISRCTN, ISRCTN13939744. Registered 9 July 2015, http://www.isrctn.com/ISRCTN13939744.

## Background

Central venous catheters (CVCs) are widely used in hospitalized patients, but are associated with infections, and mechanical and chemical complications [1, 2]. Sutures are frequently used to secure CVC onto the skin, despite not being recommended [3, 4]. Application of sutures carries a risk of injury and also disrupts the skin at the insertion site. More recently, it has been demonstrated that sutures serve as a nidus for microbial growth and become heavily colonized [5]. These micro-organisms may act as a source of catheter-related infections (CRI) including blood stream infection [6].

Alternative methods to sutures for short-term CVC securement are available. These include staples, anchor devices, catheter holders, tissue adhesives, tapes and dressings. However, evidence of their efficacy in securing a short-term CVC is limited, with the majority of available data derived from small-scale studies and clinical observations [7, 8]. Furthermore, most of the evidence is derived from peripheral, arterial and peripherally inserted central venous catheter (PICC) securement. Data on the efficacy of these devices for securing short-term CVC in various anatomical locations are also limited.

Our aim was to evaluate a new adhesive suture-free securement system and to compare it to sutures and a transparent film dressing in securing short-term CVC. The catheters were observed daily throughout their placement, and the main outcome measures were catheter migration at the skin insertion site (i.e. partial catheter dislodgement) and unplanned CVC removals (i.e. complete catheter dislodgement).

## Methods

This international, multicentre, prospective, observational, randomized, non-blinded feasibility study was carried out on four intensive care units (ICU) in three European countries. Ethical committee approval for this study was sought in each country. In the UK, approval was granted by the National Research Ethics Service Committee North West—Greater Manchester South (REC Reference 15/NW/0185), in Spain by Institut Catala de la Salut, Hospital Universitari Arnau de Vilanova, and in France by the Comité de Protection des Personnes Ouest III (Protocol # 15.04.19). The trial was registered with the International Standard Randomized Controlled Trials Number (ISRCTN 13933744).

### Study population

Between August 2015 and January 2017, adult patients on a study ICU, who required a single, short-term, non-cuffed, non-tunnelled central venous catheter (CVC), were considered for inclusion. Exclusion criteria included: confusion [patients who had a positive confusion assessment method for the ICU (CAM-ICU) score or if confusion was expected after sedation was stopped]; excessive perspiration (skin became moist within 2 min of drying); non-adherent skin, burn, trauma or other condition affecting the skin integrity at the potential CVC insertion site; uncorrected bleeding diathesis; allergy to device components; more than one catheter inserted at the same location; or pregnant or breastfeeding women. In the UK and Spain, participant written consent was obtained, and in France, a verbal no objection for study participation was confirmed as specified by local ethical approval. Patient demographics and clinical details were recorded on enrolment.

### Catheter and securement placement

**Fig. 1:**
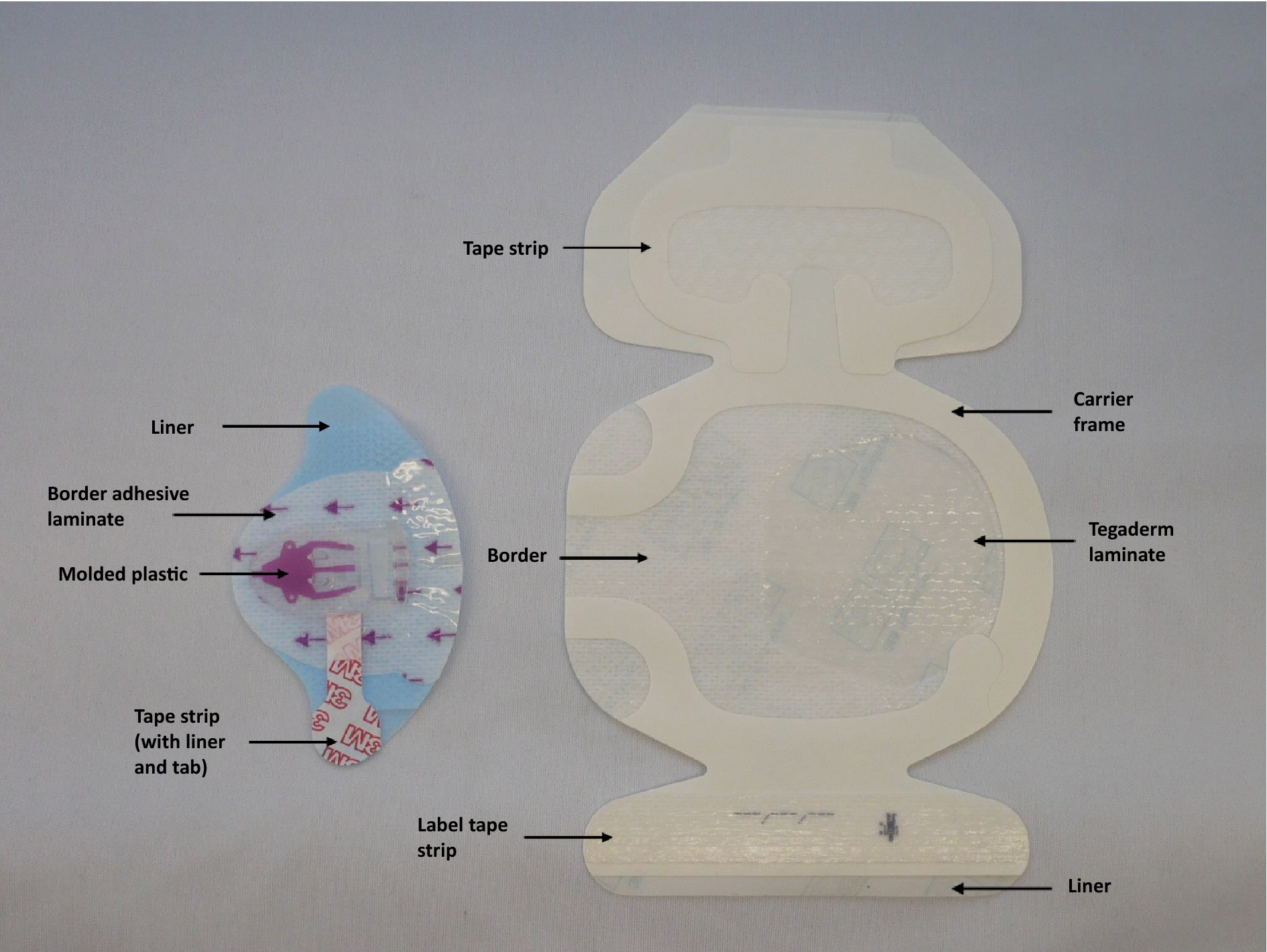
3M™ PICC/CVC Securement Device consisting of a moulded plastic device (size 5.1 cm × 5.4 cm) integrated onto a breathable base with a silicone adhesive (Tegaderm™ IV Advanced Securement device, on the left) and a soft cloth bordered transparent film dressing (Tegaderm™ IV Advanced Securement dressing, on the right; 8.5 cm × 11.5 cm)

Consented patients, who met the study criteria, were randomized to receive either a 3M™ PICC/CVC Securement Device (3M Health Care, Minnesota, USA) (Fig. 1) or sutures to secure their CVC onto their skin. A Tegaderm™ IV Advanced Securement (3M Health Care) dressing was then used to cover the CVC insertion site in both groups, and the size was recorded. The number of sutures and their application were according to local practice. The securement system and dressing were applied as per the manufacturer’s instruction. In summary, the patient’s skin was dried prior to application. Lumens of CVC were placed into the channels of the moulded plastic securement device and weaved under the plastic arm. The lumens were then secured into position with an integrated tape strip. The securement device was held in the adopted position against the patient’s skin, and the adhesive base was exposed. The dressing was applied to cover both the insertion site and the securement device. Staff received training on the application and removal of the securement device and dressing. Insertion procedure details including hair removal, site and ease of insertion and the time required were recorded. Catheter characteristics, including French gauge, the number of lumens and the type, were also noted. The time required to apply the selected CVC securement and the ensuing length of the external CVC relative to the insertion site were also determined.

### Catheter migration and unplanned/planned removals

The external length of CVC was measured daily and following dressing or securement device replacement. The measurement was taken from where the catheter entered the skin (the CVC skin entry point) to the start of the CVC hub. The daily CVC migration was calculated from transforming any measured daily migration (i.e. inward or outward CVC movement) as a positive value. The catheter migration range was deduced from the difference between the lowest and the greatest measured external CVC length for each patient. Reasons for CVC removal and any unresolved catheter occlusions and the number of patients requiring an alternative catheter securement method were recorded.

### Adverse events

Any adverse events and inoculation injuries which occurred during the CVC insertion and application of sutures were recorded.

### Securement and dressing application, removal and replacement

Daily assessment included securement device and dressing integrity and adherence onto the skin and catheter, and the number of dressing changes and reasons for replacement.

The ease of removal and replacement of the securement systems were evaluated, and the time and reasons for performing these procedures were also recorded.

### Patient and of the catheter insertion site assessments

Daily assessment included patient comfort (pain score) and sedation score, and erythema, swelling and bleeding at the catheter insertion site.

### Data analysis

Patients were enrolled into the study for the duration their CVC was in situ and remained as an inpatient. They were followed up to either CVC removal or discharge. Some patients were withdrawn from the study. Reasons for this included discharge within 24 h following CVC placement or before the first post-baseline assessment was completed. These patients were excluded from the final study analysis.

This study was designed as a feasibility study and therefore was not powered to demonstrate statistically significant differences. Patient background data were analysed with Fisher’s exact and Mann–Whitney tests. The primary outcome measure, the number of complete CVC dislodgements, and the secondary outcome measure, the number of partial CVC dislodgements, were analysed by Fisher’s exact and Pearson’s Chi-square tests.

## Results

### Patient and catheter placement characteristics

**Fig. 2:**
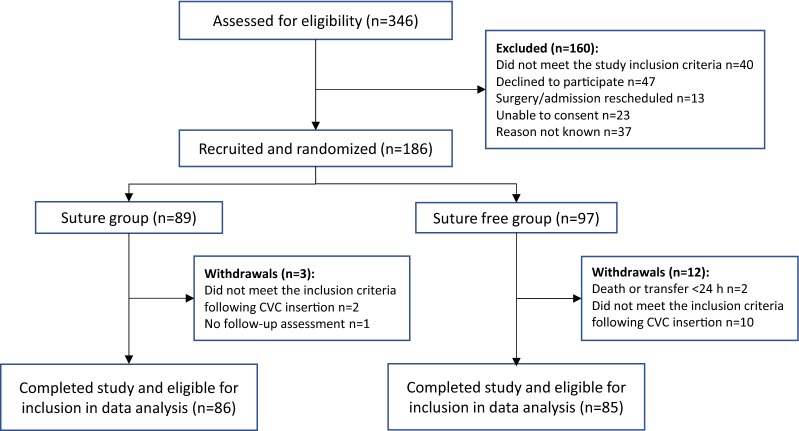
Study CONSORT flow diagram. Critical care patients in four study centres were screened and enrolled patients randomized to receive either sutures and a Tegaderm™ IV Advanced dressing (suture group) or 3M™ PICC/CVC Securement Device + Tegaderm™ IV Advanced dressing (suture-free group) to secure a short-term central venous catheter (CVC) onto the skin

The CONSORT flow diagram for screened and enrolled patients is presented in Fig. 2. In total, 186 patients met the study criteria, and were consented and randomized to receive either CVC securement method. Of these, 171 patients had at least one daily assessment recorded and were included in the data analysis. Patient and clinical characteristics were not found to be different in both groups, except for the number with a tracheostomy which was higher in the suture-free group (Table 1). The presence of a tracheostomy did not have an effect on CVC dislodgement nor daily CVC migration.

**Table 1:** Clinical and patient characteristics

	Suture group (*n *= 86 patients)	Suture-free group (*n *= 85 patients)	*p* value
Median (IQR) age (years)	66 (56.3; 74)	62 (51; 72)	0.222^a^
Median (IQR) APACHE II score	23 (15.5; 32)	21 (14; 29)	0.271^a^
Median (IQR) BMI	26.7 (23.0:31.9) (*n* = 80)	26.8 (24.3:30.5) (*n* = 73)	0.509^a^
Male-to-female ratio (%)	59:27 (68.6:31.4)	68:17 (80.0:20.0)	0.115^b^
*Number of patients (%) receiving*			
Sedative agents	38 (44.2)	47 (55.3)	0.170^b^
Paralysing agents	11 (12.8)	7 (8.2)	0.456^b^
Endotracheal tube	43 (50.0)	50 (58.8)	0.284^b^
Tracheostomy	7 (8.1)	16 (18.8)	0.046^b^
Mechanical ventilation	46 (53.5)	55 (64.7)	0.162^b^
Non-invasive ventilation	18 (20.9)	15 (17.6)	0.699^b^
Haemodialysis	9 (10.5)	15 (17.6)	0.194^b^
ECMO	2 (2.3)	2 (2.4)	1.00^b^

RASS score (%)	(*n *= 494 catheter days)	(*n *= 580 catheter days)	
1 or above	30 (6.1)	33 (5.7)	0.539^b^
0	227 (46.0)	249 (42.9)	
− 1 or less	237 (48.0)	298 (51.4)	

Patients were randomized to receive either sutures and a Tegaderm™ IV Advanced dressing (suture group) or 3M™ PICC/CVC Securement Device + Tegaderm™ IV Advanced dressing (suture-free group) to secure a short-term central venous catheter (CVC) onto the skin [*n *= number of patients or catheter days (IQR or %)]

APACHE II score, the acute physiology and chronic health evaluation score; BMI, body mass index; ECMO, extracorporeal membrane oxygenation; RASS score, the Richmond Agitation–Sedation Scale score

^a^Mann–Whitney test; ^b^Fisher’s exact test

CVC characteristics including their size, number of lumen, hair removal, anatomical location and time required for insertion were not significantly different in both groups except for the number of patients who had a box clamp (a device that grips the catheter below the hub and has flanges to accommodate sutures) which was higher in the suture group (Table 2). Box clamp use was associated with longer sections of externally protruding catheters following CVC insertion. The median external length of catheter following insertion was 11.1 mm (95% CI 5.6, 16.7) with a box clamp and 3.9 mm (95% CI 1.8, 6.0) without a box clamp (*p* = 0.012, Mann–Whitney test). The median number of sutures applied was 2.9 (95% CI 2.6, 3.9) in the 27 patients who received a box clamp as compared to 2.0 (95% CI 1.9; 2.1) in the 55 patients without a clamp (*p* < 0.0001, Mann–Whitney test).

**Table 2:** Central venous catheter (CVC) and CVC securement characteristics in the two study groups

	Suture group (*n *= 86)	Suture-free group (*n *= 85)	*p* value
Median (IQR) size (Fr gauge) of CVC	7 (7; 8.5)	7 (7; 8.5)	0.818^a^
Median (IQR) number of lumens	3 (3; 4)	3 (3; 4)	0.897^a^
Insertion site IJ:SC:FM (%)	34:28:24 (39.5:32.6:27.9)	36:25:24 (42.4:29.4:28.2)	0.895^b^
Hair removed from insertion site (%)	15 (17.4)	12 (14.1)	0.676^b^
Number of CVC with a box clamp (%)	27 (31.4)	3 (3.5)	< 0.0001^b^
Mean (95% CI) external CVC length (mm) immediately following CVC insertion^d^	6.2 (3.9; 8.5) (*n* = 84)	16.2 (10.0; 22.3) (*n* = 84)	0.299^a^
No. of catheter days with assessment (%)	532 (91.3)	643 (90.4)	0.610^c^
Mean (95% CI) duration (days) the CVC in place	6.8 (5.5; 8.1)	8.4 (6.6; 10.1)	0.162^a^
Median (IQR) time (min) to apply CVC securement	3 (2; 6.5) (*n* = 79)	5 (3; 5) (*n* = 78)	0.184^a^
Median (IQR) time (min) to replace CVC dressing/securement device	7 (5; 10) (*n* = 90)	10 (5; 10) (*n* = 129)	0.006^a^
Median (IQR) time (min) to remove CVC dressing/securement device	3 (2.5; 5) (*n* = 25)	2 (1; 4) (*n* = 29)	0.08^a^

Patients were randomized to receive either sutures and a Tegaderm™ IV Advanced dressing (suture group) or 3M™ PICC/CVC Securement Device + Tegaderm™ IV Advanced dressing (suture-free group) to secure their CVC onto the skin [*n* = number of CVC or CVC days (IQR or %)]

IJ, internal jugular vein; SC, subclavian vein; FM, Femoral vein

^a^Mann–Whitney test; ^b^Fisher’s exact test; ^c^Pearson’s Chi-square; ^d^external CVC length measured in millimetre from the CVC skin entry point to the moulded junction of the CVC hub

### Catheter migration and unplanned/planned removals

Overall, there was no significant difference in CVC migration between the two securement systems, with 41.9% (36 out of 86) of CVC secured with sutures and 56.5% (48 out of 85) of CVC secured with suture-free securement system having CVC migration of ≥ 2 mm (*p* = 0.056; Pearson’s Chi-square) (Table 3).

**Table 3:** Migration of short-term central venous catheters (CVCs) when secured with sutures and a Tegaderm™ IV Advanced dressing (suture group) or 3M™ PICC/CVC Securement Device + Tegaderm™ IV Advanced dressing (suture-free group) [*n* = number of observations or CVC (%)]

Movement in millimetre	Suture securement group [*n* = no. of observations or CVC (%)]	Suture-free securement group [*n* = no. of observations or CVC (%)]	*p* value (Pearson’s Chi-square)
*Daily movement*	No. of observations (*n* = 523)	No. of observations (*n* = 624)	
0–1	455 (87.0%)	539 (86.4%)	0.686 (1.5)
2–5	55	63	
6–10	8	11	
> 10	5	11	
*Movement due to replacement*	No. of replacement (*n* = 84)	No. of replacement (*n* = 114)	
0–1	67 (79.8%)	96 (84.2%)	0.366 (3.2)
2–5	17	13	
6–10	1	3	
> 10	1	2	
*CVC movement range*	No. of CVC (*n* = 86)	No. of CVC (*n* = 85)	
0–1	50 (58.1%)	37 (43.5%)	0.146 (3.9)
2–10	27	38	
> 10	9	10	

Unplanned removal of a CVC occurred in 2 out of 86 (2.3%) patients in the suture group and 5 out of 85 (5.9%) patients in the suture-free group (*p* = 0.277, Fisher’s exact test). Four CVC in the suture group (days 5, 7, 12 and 14) and five CVC in the suture-free group (days 8, 35 and three on day 11) were removed due to fever or suspected infection which were not subsequently confirmed as being CVC related. Occlusion or non-functional CVC led to CVC removal on one occasion in both securement groups. In the suture-free group, thrombus formation in one catheter resulted in the CVC being removed.

### Subgroup exploratory multivariate analysis

Exploratory multivariate analysis was carried out on the daily CVC migration data. Significantly less daily CVC migration was demonstrated in the suture group when a box clamp was used [6.5% of the CVC daily movement recorded ≥ 2 mm (9 out of 139 observations)] as compared to no box clamp application [15.4% of the CVC daily movement recorded ≥ 2 mm (59 out of 384 observations); *p* = 0.008, Fisher’s exact test].

CVC migration was also more prevalent when a 4-lumen CVC was inserted and secured with sutures in comparison with a 3-lumen CVC secured with sutures [36 out of 179 (20.1%) vs. 31 out of 335 (9.3%) daily observations recorded movement ≥ 2 mm, respectively; *p* = 0.001]. However, in the suture-free group the difference in the frequency of CVC migration between a 4-lumen and 3-lumen CVC was not significant [16 out of 149 (10.7%) vs. 68 out of 473 (14.4%) daily observations recorded catheter movement ≥ 2 mm, respectively; *p* = 0.275].

### Adverse events

One adverse event was recorded in the suture-free group, when a catheter became twisted. Two incidences of needlestick injury occurred in one study centre during CVC insertion, both in the suture securement study group.

### Securement and dressing application, removal and replacement

There were no significant differences in the mean number of dressings used per CVC, with 2.24 (95% CI 1.93, 2.56) and 2.73 (95% CI 2.29, 3.17) in the suture and suture-free groups, respectively (*p* = 0.109, Mann–Whitney test). The causes for dressing replacement in the suture and suture-free group were also comparable, with 27.4% and 28.0% of replacement being scheduled and 31% and 25.5% of replacement due to excessive dressing lift, respectively. The suture-free securement device had to be repositioned on two occasions (two separate patients) due to incorrect application of the securement system. Two patients (2.3%) had sutures which became detached during the study, and the dressing became completely detached on two occasions (in separate patients). The suture-free securement system was replaced in three occasions (in separate patients) due to system detachment.

The time to apply and remove sutures and a dressing or a suture-free securement device and a dressing were not significantly different (Table 2). However, the time required to replace the dressing plus suture-free securement device was longer than replacement of the dressing in the suture group (*p* = 0.006, Mann–Whitney test). Suture securement application was rated easy or reasonable in 54 out of 55 (98.2%) applications assessed compared with 48 out of 54 (88.9%) suture-free securement system applications (*p* = 0.048, Fisher’s exact test). Removal was rated easy or reasonable in 24 out of 25 (96%) of suture securement removals and 28 out of 29 (96.6%) of the suture-free securement system removals. Dressing replacement in the suture group was rated easy or reasonable in 99 out of 100 (99%) user assessments in comparison with 117 out of 131 (89.3%) assessments of the suture-free securement device (*p* = 0.002, Fisher’s exact test).

### Patient comfort and assessment of the catheter insertion site

The assessment of patient pain score during the dressing and securement system removal or replacement was not significantly different in both study groups (*p* = 0.192, Kruskal–Wallis test). In addition, the daily observation for erythema under the dressing was not scored differently in both groups. Seventeen out of 86 patients in the suture group and 19 out of 85 patients in suture-free group patients had erythema around the CVC insertion site (*p* = 0.711, Fisher’s exact test). Similarly, the other daily assessments of the CVC insertion sites, including swelling (3.5% vs. 1.2%), bruising (5.8% vs. 3.5%), or discharge (37.2% vs. 47.1%) whilst the CVC were in situ, were not significantly different between the two securement methods. However, 20.9% of the patients had erythema around the sutures and 9.3% of the patients had blood and or serous fluid discharge at the suture site.

## Discussion

In this feasibility study, the new suture-free securement for short-term CVC performed satisfactorily as compared to sutures. The overall net migration of the CVC was not significantly different in the study population. In addition, unplanned removal of the CVC was < 6% in both groups. Although the study was not statistically powered and further clinical evidence is required to evaluate the suture-free securement performance on a wider group of patients, this is encouraging data on alternative CVC securement methods to sutures. The suture-free devices have been designed foremost to reduce the risk of inoculation injury, by reducing the need for using sharps in the clinical environment. In the USA, 26% of percutaneous injuries were caused by suture needles [9]. Although the current study was not powered to study this relationship further, it is accepted that suture-free devices will reduce the risk of inoculation accidents and transmission of blood-borne pathogens, such as HIV and hepatitis B and C. This is reflected in current recommendations [4].

Moreover, this evaluation is the first clinical study, to our knowledge, where daily migrations of short-term CVC relative to the insertion site in critical care patients were assessed. We report that migration of short-term CVC occurs frequently, although the majority of the movement did not result in discontinued use of the CVC or increased reports of adverse events. However, these small catheter movements occurred both inwards and outwards relative to the skin, which may facilitate the introduction of micro-organisms located at this site. This is consistent with the recognized risk of CRI originating from micro-organisms at the CVC insertion site [10] and the recommended use of chlorhexidine dressings to decrease this microbial load [3, 11]. CVC migration was most likely related to the relative movements of the patient and the overall weight of the external components of the CVC, as was observed with 4- compared with 3-lumen CVC secured with sutures. These study findings support current guidance which recommend use of a CVC with the minimum number of lumens and assessing the individual patient’s risks and benefits when selecting the CVC insertion site [3, 12]. Although improved stabilization of CVC was achieved with a box clamp and additional sutures, which were applied to over 30% of the CVC in the suture group patients, the increased risk of both microbial contamination and inoculation injury needs to be considered against the alternative securement methods available for a short-term CVC. Suture-free CVC securement methods need to exhibit competitive benefit–risk profiles to sutures by offering a safe and appropriate level of patient care whilst meeting securement performance needs. In addition to the comparable catheter securement between the two securement systems, skin reactions including erythema and swelling at the insertion site as well as the patient comfort level during dressing and securement system removal or replacement were also similar. The time required for applying and removing sutures and a dressing or suture-free device and a dressing did not vary significantly. However, additional time was required to replace the suture-free securement device and a dressing. The ease of replacing the suture-free securement system was rated worse than replacing the dressings only in the suture group. Securement of CVC with sutures and a transparent film dressing is a well-established practice, and the lack of experience in using the new type of CVC securement method, as well as the need for replacing both the dressing and a device, may have influenced these results. It is however likely, that with experience, the time to replace the new type of securement system will reduce. The acquisition cost of the suture-free securement system (adhesive securement device plus a dressing) ranges between €6 and €11, depending on the type and size of the dressing, in comparison with €1 to €6 for the dressing alone. However, to provide an accurate cost comparison between the two securement methods, additional equipment including sterile forceps for the application of sutures, sterile scalpels for removal, and potentially local anaesthetic agent would also need to be taken into account.

In addition to the small number of patients studied, another limitation of this study was it only included ICU patients and excluded confused, excessively perspiring or bleeding patients who are potentially at greater risk of CVC migration and dislodgement. Patient mobility including movement in bed or walking may also influence the CVC securement and risk of unplanned removal of CVC. However, in this study no significant differences in CVC migration were found between patients with different levels of consciousness. We therefore considered that the selected study group of patients who were closely monitored was suitable for the initial assessment of the new suture-free device. Further clinical studies are, however, required to assess the suture-free device outside the critical care and also to broaden the patient inclusion criteria.

## Conclusions

This study demonstrated frequent migration of short-term CVC in relation to the skin insertion site. The new suture-free CVC securement system, evaluated on selected patient group in ICU, was comparable to sutures in securing CVC. Removing the need for sutures for adequately securing CVC may reduce the likelihood of inoculation injuries to staff and potentially decrease the microbial load around the CVC insertion site. Further clinical studies are needed to evaluate the suture-free CVC securement system in a wider range of patient groups, including those in the non-critical care environment.

## Abbreviations

CAM-ICU: confusion assessment method for the ICU
CVC: central venous catheter
ICU: intensive care unit
ISRCTN: International Standard Randomized Controlled Trials Number
PICC: peripherally inserted central venous catheter
